# Editorial: Technological innovations to address social isolation and loneliness in older adults

**DOI:** 10.3389/fpubh.2023.1139266

**Published:** 2023-02-06

**Authors:** Hongtu Chen, Sue E. Levkoff, Helianthe Kort, Quentin A. McCollum, Marcia G. Ory

**Affiliations:** ^1^Department of Psychiatry, Harvard Medical School, Boston, MA, United States; ^2^College of Social Work, University of South Carolina, Columbia, SC, United States; ^3^Department of the Built Environment, Eindhoven University of Technology, Eindhoven, Netherlands; ^4^Environmental and Occupational Health, School of Public Health, Texas A&M University, College Station, TX, United States

**Keywords:** technology, loneliness, social isolation, aging, global, pandemic

Social isolation and loneliness are widely recognized as a global challenge for population aging (1). Mounting evidence has shown that both social isolation and loneliness are associated with increased risks of major physical, cognitive, and psychological morbidities as well as lower perceived wellbeing and health-related quality of life among older adults (2).

Among various policy and social service delivery approaches, innovative technologies have emerged as a promising solution to reduce social isolation and loneliness for this population, and/or to increase support to family members of older adults in need (3, 4).

While our research community continues to face the challenges of finding feasible and cost-effective solutions to improving social connection and support for older adults, the historical and global context of the COVID-19 pandemic has further highlighted the advantages of using digital technology to assist vulnerable populations such as older adults in both residential homes and institutional settings.

This Research Topic includes over 20 contributions from authors all over the world. Their papers represent the frontiers of the interface between the challenges of the need to address social isolation and loneliness among older adults and the research opportunities associated with the advancement of digital technologies. Several themes are emerging as summarized below.

## 1. Mobile technology use reduces loneliness

Mobile technologies have been instrumental in transforming the way in which older adults interact with each other, find information, and access resources to improve their health and wellbeing (5). Behaviors such as making video calls, participating in social media, or simply using the internet to search for information, can help improve social connection, reduce loneliness, and attain a higher quality of life.

In a narrative review of the literature describing the use of video calls in geriatric institutions between 2000 and 2021, Naudé et al. from France reviewed 15 studies focusing on the usability, acceptability, and effectiveness of video calls, and conducted a qualitative, deductive thematic analysis inspired by a Health Technology Assessment (HTA) multidimensional model. They found encouraging evidence for the feasibility of video call use in geriatric institutional settings, and its efficacy in reducing social isolation among residents, while at the same time, identifying numerous technical, human-related, ethical, and organizational barriers to their use.

Using data from the 2016 wave of the US Health and Retirement Study, Byrne et al. analyzed the self-reported frequency of social technology use (e.g., communication through Skype, Facebook, or other social media with family and friends). They found that social technology use is less prevalent among rural older adults than urban and suburban-dwelling older adults. Among rural older adults, those who use social technology less frequently experience greater loneliness than urban older adults, suggesting the importance of considering rural disparities in designing social technology interventions for older adults.

Czaja et al. from the US analyzed the baseline data from a sample of older adults who participated in an intervention trial that examined the beneficial effects of a software system designed to both support access to resources and information and social connectivity. They found that loneliness was strongly associated with depression and self-ratings of health, and that having a smaller social network, more functional limitations, and limitations in engaging meaningful activities were associated with higher levels of loneliness and greater social isolation. They posit that information and Communication Technologies (ICTs) can be used to promote social connectivity and engagement.

Using a larger sample of older adults who participated in the China Longitudinal Aging Social Survey, Li et al. found that only a small percentage of older Chinese adults often used the Internet to engage in at least one activity, and that internet users were less likely to be depressed and had a higher level of cognitive function. They also found that among those who used the Internet more, the depression levels of socially isolated male participants were much lower than female participants, suggesting protective impacts of internet use.

Using data from one region in East China, Yang et al. examined the factors influencing the digital inclusion of older adults and the relationship between digital inclusion and quality of life. They found that attitudes toward technology were the most significant factors predicting their digital inclusion, and further, that digital inclusion was associated with higher quality of life among Chinese older adults. This research confirms the importance of ICT as an important pathway for wellbeing among older adults.

Using data from the China Longitudinal Aging Social Survey (CLASS), Xie et al. analyzed the effects of Internet use on the mental health of Chinese older adults and found that internet use increases depressive symptoms in older adults and that depressive symptoms are associated with female gender, younger age, high-income, non-rural residence, less educated, and living with others. They speculate that internet use may reduce actual in-person human relationships or actual social interaction. The discussion reinforces the long-standing caution for prudence in assessing and weighing the benefits and detriments brought about by new technology.

## 2. How technologies help those with cognitive impairments

While applauding the emergent positive impacts of ICT in reducing social isolation for older adults, it is also important to understand how technologies can benefit those with cognitive impairments and their caregivers (6, 7). Several research teams explored how ICT can help the caregivers of persons with dementia by increasing access to learning opportunities for knowledge and skills necessary to improve their capacity to care for both patients with dementia and themselves. In general, more research is needed to assess how technologies can assist those patients with varying levels of cognitive impairments.

Prophater et al. reported the effectiveness of an 11-month intervention in preventing social isolation and increasing mood among older adult residents of senior care communities (e.g., assisted living communities, and skilled nursing communities) during the COVID-19 pandemic in the US. The intervention included distributing personalized Wi-Fi-enabled iN2L tablets to the senior care communities to connect and engage residents and their families and providing a video-based learning platform. A survey of program staff indicated that residents struggled with loneliness and mood and that the tablet was useful in improving loneliness and mood and reducing risks of dementia or cognitive decline in residents, and allowing them to stay in touch with family and friends.

In a scoping review, Huisman et al. from the Netherlands showed that technology applications that target caregivers of persons with dementia can both lower caregiver burden and/or improve caregiver quality of sleep and reduce social isolation. Of interest, interventions may target the person living with dementia, their informal caregiver, or both.

Lydon et al. from the US conducted a narrative review of the research on social engagement in persons with mild cognitive impairment (PwMCI). They found that PwMCI may have different levels of social engagement than those experiencing typical cognitive aging, and in-person social engagement can have a positive impact on cognitive, emotional, and physical health for PwMCI. They note that very few intervention studies have targeted social engagement, but both in-person and technology-based interventions appear to have promising health and wellbeing outcomes.

## 3. New technologies beyond ICT for older adults at home

Digital technology not only has enhanced convenience and affordability for social connection, it also provides an array of new opportunities to enrich the daily lives of older adults (8). Through the examples of voice-control devices, games, or home-based sensor systems, we are peeping into a future where artificial intelligence (AI), internet of things (IOT), and ambient and wearable sensor technologies are increasingly interwoven with human behavior, offering new possibilities to enable the older adults with disabilities and functional limitations.

In a study aiming to understand the influence of personal voice assistants (PVA) on loneliness reduction among adults of advanced ages, Jones et al. from the US assessed 16 older adults using an Amazon Echo PVA for 8 weeks. They found that after the first 4 weeks of the intervention, participants reported significantly lower loneliness, and that relational greetings (i.e., user-initiated, friendly phrases) predicted loneliness reductions in the first 4 weeks and baseline loneliness predicted relational greetings with the PVA during the entire 8 weeks.

Corbett et al. from the US reviewed older adults' use of commercially available artificial intelligent virtual home assistants (VHA) (e.g., Amazon Echo, Google Nest) and found that VHAs are perceived by many older adult users as “companions” and helpful for improving social connectedness and to reduce loneliness. Further research needs to address privacy concerns and other ethical issues as well as costs associated with VHA use as potential barriers to older adults' VHA adoption and use.

In assessing older adults' perception of two commercially available exergames, Freed et al. from the US found that greater enjoyment and the greater likelihood of future play were significantly related to a relatively younger age. Participants were highly motivated to do well on the games but reported lower scores for the likelihood of playing these games in the future. The preliminary results of this pilot study suggest that exergames may help address social isolation and loneliness.

A 12-month observational study in Switzerland evaluated a new in-home monitoring system that continuously monitored older adults' daily activities (e.g., mobility, sleep habits, fridge visits, door events) by an ambient sensor system and health-related events by wearable sensors. Pais et al. found that the majority of older adults, family caregivers, and support nurses reported that in-home sensors helped with staying at home, improved home care and quality of life, prevented domestic accidents, and reduced family stress.

## 4. Toward building a better community

The advancement of digital technology has helped older adults get better connected with their family members and friends, and also gradually changed many older adults' home environment, reflecting the WHO's call for more age-friendly environments in the UN Decade of Healthy Aging (9). Thus, some investigators explored the notion of a better neighborhood or community designed to bring broader social resources (e.g., volunteers, paid workers, and other “strangers”), *via* the advantage of technology, for the benefit of older residents.

Sandu et al. from the US regarded a good neighborhood as one that addresses loneliness and barriers to care faced by vulnerable populations such as older adults. They instituted a Good Neighbor Program of weekly phone calls conducted by student volunteers to community-dwelling older adults throughout the course of 1 year. The program not only provided another layer of support to identify and refer issues in older adults, it also had positive impacts on the caller.

The smart city agenda has attempted to bring about technological change whilst also improving access to urban resources for aging well. Li and Woolrych conducted a qualitative study with older people across three diverse neighborhoods in the city of Chongqing, China. They explored the experiences of older people living in a smart city in China to examine how the smart city and age-friendly agenda can be brought together to support positive social outcomes for older people. They identified the potential for improved health and wellbeing and social connectedness while identifying challenges such as widening social inequalities, issues of safety and security, and exclusion from the co-production of smart city policy and practice.

Paid and unpaid caregivers may respond differentially to the use of technology. In a nationwide survey of caregivers, Lee et al. from the US examined the association between communication technology use, perceived social support, and sense of belonging. They found that the use of communication technology was associated with an increased sense of belonging to their local community among paid caregivers, yet did not contribute to feelings of belonging among unpaid caregivers. Further research is needed to understand the effectiveness of different digital technology interventions in both populations.

An information and communication technology (ICT) training program is a promising strategy to reduce social isolation and loneliness for homebound older adults. Jiménez et al. from the US found that it is important to identify successful strategies for recruiting both volunteers and participants, to incorporate flexibility when delivering interventions to homebound older adults, and to monitor the participant-volunteer relationship through volunteer-completed reports to mitigate barriers to the successful implementation of the ICT training program.

## 5. Roles of technology during COVID-19 pandemic

The solicitation of articles for this Research Topic happened to fall into a special period of time when all of us, including researchers and older adults as well as their immediate supporters, were affected by a global social and health crisis caused by the COVID-19 pandemic (10). The pandemic not only resulted in significant disruption to the daily living of many people, leading to potential exacerbation of their physical and emotional distress, but it also increased the number of older adults who were socially isolated as many countries issued stay-at-home orders and numerous social-distancing measures (11). Several articles highlighted the positive roles of mobile technology or technology-enabled services in helping older adults through the very challenging period of the pandemic.

The COVID-19 pandemic interrupted both life and research. In examining the effects of a 12-week therapist-supported multi-component mobile app-delivered intervention among middle-aged and older adults, Gould et al. found those who were enrolled prior to the COVID-19 pandemic experienced a significant increase in mental health quality of life (QoL) and a decrease in loneliness during the intervention; those enrolled after the pandemic began experienced a comparable increase in mental health QoL, while the decrease in loneliness during the period of mandated isolation did not hold.

The COVID-19 pandemic also led to a shift in some service programming from onsite to virtual. Sanchez-Villagomez et al. in the US noted that a hospital-based education program that used varied online approaches reached a substantial increase in program reach between April and August of 2020. Most participants reported a gain in knowledge and self-management skills and that virtual programming helped to foster social connectivity, helped to build a daily routine, and positively impacted mental and physical health despite the quarantine orders.

In a Canadian survey of older adults during the COVID-19 pandemic, Horst et al. found that many older adults felt isolated in 2020, regardless of most demographic factors (e.g., age, gender, education, disability) that were previously associated with increased isolation risk. Given that technology proficiency was seen as an independent, modifiable factor in reporting less isolation, future efforts to contain social isolation should consider training programs for older adults to improve technology confidence, especially in an increasingly digital world.

Strict measures practiced during the COVID-19 pandemic, such as preventing family members from visiting nursing homes for several months, were likely to have enhanced feelings of loneliness and isolation in LTC residents. Given that ICT use has been shown to help older adults maintain social interaction, Gallistl et al. from Europe argue for policy recommendations to enhance and support LTC residents' digital engagement.

In fall 2022, the WHO predicted that “the end of the COVID-19 pandemic is in sight” (12), though there are still over half a million new cases every day in the world, and another Winter is approaching signaling a potential new surge (13). Despite the growing appreciation of digital technology and virtual social contacts we have experienced over the past 3 years, we know that for many older adults, the feeling of loneliness and the fact of isolation from one another will continue. So will our optimism in believing that progress will eventually be made through scientific efforts by our resilient research community and dedicated public health practitioners across the globe.

## Author contributions

HC and SL conceived the original idea and contributed to the development of the first draft. SL and MO edited multiple versions of the manuscript. All authors critiqued the subsequent drafts. All authors contributed to the article and approved the submitted version.
